# On genomic DNA paradigms, research publications, and scholarly inquiry

**DOI:** 10.1002/humu.21353

**Published:** 2010-09-07

**Authors:** Mark H Paalman, Richard GH Cotton, Garry R Cutting

**Affiliations:** 1 Managing Editor, Human Mutation; Wiley-Blackwell Life Science Journals, John Wiley & Sons, HobokenNew Jersey USA; 2 Co-Editor, Human Mutation; Director, Genomic Disorders Research Centre, St. Vincent's HospitalMelbourne, Australia; 3 Co-Editor, Human Mutation; MuKusick-Nathans Institute of Genetic Medicine, Departments of Pediatrics and Medicine, The Johns Hopkins Medical InstitutionsBaltimore, Maryland, USA

An article published in *Human Mutation* by Gottlieb et al. [2009] received much attention for the reported observation of tissue-specific polymorphic differences in the *BAK1* genotype among abdominal aortic anerurysm (AAA) patients and some controls. In particular, sequencing revealed a statistically significant number of instances in which single nucleotide polymorphisms (SNPs) present in tissue samples from patient abdominal aorta (AA) were absent from matching blood samples. The authors proposed a novel hypothesis “postulating that multiple variants of genes may preexist in ‘minority’ forms within specific non-diseased tissues and be selected for, when intra- and/or extra-cellular conditions change” [Gottlieb et al., 2009].

The implications of this unusual finding are of potential significance as regards the role somatic mutation and intraorganismal selection might play in genetic disease, not to mention the suitability of using blood-derived genomic DNA to evaluate patient mutational status of genetic diseases that affect various tissues. The work was challenged in a Letter to the Editors by Hatchwell [2010], who argued that the Gottlieb et al.'s [2009] findings were likely artifactual due to pseudogene interference, genomic DNA contamination of RNA samples, and inaccurate reference sequences in GenBank. Gottlieb et al. [2010a] responded to this criticism with additional information about the methods they used, both in vitro and in silico, to reduce the possibility for such artifacts.

In the current issue, Küry et al. [2010] discuss additional concerns that they have about the original article in another letter. These concerns have, in turn, been responded to by Gottlieb et al. [2010b].

These four letters were all carefully peer reviewed and evaluated editorially, but within the limitations of the rather constrained “Letter to the Editors” format. As the Managing Editor and Co-Editors of *Human Mutation*, we feel that discussion of the original article by Gottlieb et al. [2009] and its implications is valuable for the scientific process. At this juncture, however, we believe that further comment on this study must be accompanied with thorough, original research aimed to either validate or refute the original findings. We welcome such submissions for editorial consideration and peer review.
